# Enhancing the African bioethics initiative

**DOI:** 10.1186/1472-6920-4-21

**Published:** 2004-10-15

**Authors:** Temidayo O Ogundiran

**Affiliations:** 1 Division of Oncology, Department of SurgeryCollege of Medicine, University of Ibadan and University College Hospital, PMB 5116 Ibadan, Nigeria

## Abstract

**Background:**

Medical ethics has existed since the time of Hippocrates. However, formal training in bioethics did not become established until a few decades ago. Bioethics has gained a strong foothold in health sciences in the developed world, especially in Europe and North America. The situation is quite different in many developing countries. In most African countries, bioethics – as established and practiced today in the west- is either non-existent or is rudimentary.

**Discussion:**

Though bioethics has come of age in the developed and some developing countries, it is still largely "foreign" to most African countries. In some parts of Africa, some bioethics conferences have been held in the past decade to create research ethics awareness and ensure conformity to international guidelines for research with human participants. This idea has arisen in recognition of the genuine need to develop capacity for reviewing the ethics of research in Africa. It is also a condition required by external sponsors of collaborative research in Africa. The awareness and interest that these conferences have aroused need to be further strengthened and extended beyond research ethics to clinical practice. By and large, bioethics education in schools that train doctors and other health care providers is the hook that anchors both research ethics and clinical ethics.

**Summary:**

This communication reviews the current situation of bioethics in Africa as it applies to research ethics workshops and proposes that in spite of the present efforts to integrate ethics into biomedical research in Africa, much still needs to be done to accomplish this. A more comprehensive approach to bioethics with an all-inclusive benefit is to incorporate formal ethics education into health training institutions in Africa.

## Background

Before the modern discipline of bioethics evolved, ethics had been on the centre stage of medical practice for more than two millennia, since the time of Hippocrates. In 1803, Thomas Percival published his book on *Medical Ethics*, which became the template on which the code of ethics of the American Medical Association was based in 1847 [1]. Medical ethics then existed as a code of conduct for medical practitioners and was aimed at the physician putting the interests of his patients uppermost at heart. However, the origin of bioethics, as it is known and practiced today, can be traced back to three different but interrelated events: a set of scandals in biomedical research, advancement in medical technology and the civil rights movement [2]. Of the scandals, the most well-known is the infamous Nazi experimentation on war prisoners and the subsequent Nuremberg trials of the 1940s. As a result of this and other scandals that trailed the medical profession subsequent to Nuremberg, various codes, declarations, guidelines, policies and documents came into existence and became widely applied to research with human subjects and to health care practice as a whole. The scandals and the events thereafter led to three developments. First, physicians became more sensitive to the ethics of the profession. Medicine is a moral discipline but the indirect method of achieving moral acculturation would no longer be sufficient to equip the physician to meet the ethical challenges of modern day biomedical practice. The need for formal education in medical ethics was thus acknowledged. Second, society became sensitized to the necessity of becoming involved in the decisions which ultimately affect their health and liberty. This signaled the decline of paternalism and the rise of liberalism, individual rights and autonomy in medical practice. Third, the coming of other professionals from different disciplines like social sciences, humanities and the law into what had hitherto been the exclusive domain of medical professionals led to the "socialization" of biomedical science, which further spurred on the bioethics movement.

The scope of bioethics has continued to expand in response to changes in societal dynamics, medical technology and health care practices. Perhaps more because of its antecedents than for want of content, most of the discourse and writings in the early days of bioethics centred on issues of patient-physician relationship, respect for person, best interest of the patient, and justice in health care delivery. Today, traditional bioethics discussions and literature are changing as new ethical concerns evolve around dilemmas posed by new technology on the subjects of end of life issues, organ donation, human reproduction and human genomics. Moreover, the bioethics agenda has expanded to include the subjects of resource allocation, organizational ethics and public health ethics, among others.

Bioethics in its present form is rooted in and largely dominated by western culture. The tempo and content of bioethics discourse are largely influenced by the technological creations of the developed world. However, ethics is not exclusively the domain of the west. Core ethical values are essentially the same for all human communities leaving aside each community's customs, culture and preferences [3]. According to Potter, bioethics is "the application of ethics to all of life" [4]. In the globalization of bioethics, different cultural, ethnic and religious perspectives are given a voice. Though bioethics has come of age in the developed and some developing countries, it is still largely "foreign" to most African countries. It is time Africa joined the bioethics bandwagon. Its relevance and applications to science and research are vital and should not be overlooked. The call of this paper therefore, is for bioethics to be integrated as a required component of medical education curriculum in Africa.

## Discussion

### Research ethics

In the bioethics literature, bioethical discourse and arguments have been most prominent and intense concerning research involving human participants. One major achievement in this regard is the creation of an oversight body that sees to the proper design and conduct of research that conforms to generally acceptable and established ethical guidelines. There resides in this body the duty of ensuring that research sponsors and investigators abide by established conventions for carrying out clinical research. They also perform the role of assuring the safety of research participants and ensuring that participants (and/or society) benefit from the outcome of research. The relevance of this body to modern day health care and research is partly exemplified in the absence of any major scandals in the form and magnitude of those already recorded in history. The establishment of research ethics boards has not solved all the ethical problems of biomedical research though. There is still a lot to do to re-structure, re-empower and re-position the board to match the complexities of the present-day technology-driven medical research and practice.

#### Current efforts in Africa

Various bodies within and outside Africa have pioneered the movement towards ensuring that medical research in Africa conforms to international ethical guidelines. This is the aspect of bioethics that is most visible in Africa and has been anchored partly by the Pan African Bioethics Initiative (PABIN), a pan-African organization that was established in 2001 to foster the development of bioethics in Africa with a particular focus on research ethics (5). This idea has arisen in recognition of the genuine need to develop capacity for reviewing the ethics of research in Africa. It is also a condition required by external sponsors of collaborative research in Africa. Ethics workshops and conferences have been held in different parts of Africa, including Tanzania [6], Zambia [7], South Africa [8], Ethiopia [9], Cameroon [10] and Nigeria [11,12]. Moreover, some institutions and research centres have established research ethics review committees and some members of these committees have attended training workshops on research ethics.

While the present efforts and achievements are commendable, much still needs to be done for the effects to filter through to the grassroots, which is the main arena of research activities and where the burdens of research are most felt. I say this for the following reasons. First, the present efforts are still limited in extent and effect. Hitherto, most of the conferences have taken place in two or three geographical zones of the continent and have been limited to a few days of activities. Of course, the interests and motives of the sponsoring agencies together with the presence on the ground of those who are available to organize the conference locally determine where, when, for how long and the number of participants in the workshop. There is thus restriction on the number of researchers who can attend the conferences from all over Africa and on the amount of knowledge that can be imbibed in those few days. The consequence is that the same few people attend the conferences most of the time. These attendees from a few centres might not be able to change unethical research practices in their countries. Attempts by these few to train their colleagues locally are often constrained by lack of funds.

Second, absence of national directories of research activities in most African countries makes the magnitude of biomedical and social sciences research in Africa to be underestimated. For example in Nigeria, five categories of research and researchers are easily identifiable. Individual or institution-supported research is done by students, clinicians (including resident doctors) and other scientists. This category constitutes a significant proportion of research in tertiary academic and health institutions. Industry-sponsored research is undertaken by researchers for pharmaceutical companies to promote new or old drugs. Such research protocol may be indigenously developed or be a part of multicentre trials. In most instances, these companies do not go through the institutions where the researchers are based, but deal directly with individual researchers, who may or may not subject the research protocol to an ethics board review. Collaborative research with colleagues from the developed countries is often externally funded. It includes hospital and community-based trials and mostly involves experimenting with drugs or vaccines. Of particular ethical concern in collaborative research is the fact that external sponsors may differ in their motives for conducting research and there may be limited applicability of research benefits to the country or local community [13]. Moreover, the clinician/researcher and/or institutions are themselves vulnerable to funding pressures. Another category of research is that which occurs through indigenous government-funded agencies. An example of such an agency is the National Institute for Medical Research, which has been carrying out research in Nigeria for more than thirty years on parasitic, infective and non-infective diseases. Non-Governmental Organizations (NGOs) are also involved to variable extents in both clinic and community-based research.

Third, a majority of Africa's research participants are highly vulnerable given their low level of formal education and the political, social and economic milieu in which they live. The fourth reason is that Africa is a pluralistic society with diverse peoples and cultures. While general guidelines may apply in most cases, in some the peculiarities of each ethnic and cultural group will significantly affect what research is done and how it is done in those communities. Lastly, not every research centre has established an ethics review process. Where already established, most of the ethics review committees are grossly underfunded and unequipped for their duties.

### Clinical ethics

This is the branch of bioethics that addresses ethical conflicts that arise in daily clinical practice in health care institutions, through the establishment of hospital ethics committees and ethics consultation services. Fletcher and Siegler define ethics consultation "as a service provided by an individual consultant, team or committee to address the ethical issues involved in a specific clinical case. Its central purpose is to improve the process and outcomes of patents' care by helping to identify, analyze, and resolve ethical problems" [14]. An ethics consultation service also responds to conflicts that arise from technological improvements in medical care and the increasing pressure to meet a perceived standard of care [15]. Clinical ethics is also concerned with organizational ethics and networks and the implications of health care policy at the bedside. Although there are contrasting views about the presence of ethics consultants at the bedside, hospital ethics committees are now available in most hospitals in North America and Europe, providing services to patients, families, staff and the entire hospital organization.

Do we need the services of hospital ethics committees or consultants in African hospitals or at the bedside? That may not be the point presently. However, clinical ethics is neither about committees and consultations, nor about technology and end-of-life issues alone. Common sense and intuition are insufficient to address all ethical issues that arise in patients' care. The well-intentioned clinical decisions and judgments of yesterday may turn out to be unsound in the searchlight of today's ethical scrutiny. Although core moral virtues have generally guided medical practice in Africa as elsewhere, there is the increasing need to apply both cognitive and behavioural ethical values to everyday decision making at the bedside by the physician as well as other health-care professionals [16]. Perhaps ethical concerns have unwittingly been unrecognized, downplayed or overlooked by health care professionals. The management of chronic diseases like HIV/AIDS and cancer, the incidences of which are on the increase, has attendant ethical implications about care, cost and consequences on patients' personality, values and families. Besides, Africa will someday cross the technological divide that will make resolution of ensuing ethical issues urgent, which health care providers will no longer be able to ignore. The societies are becoming more enlightened and it may be sooner than anticipated when physicians and other health care workers begin to grapple with some ethical challenges for which they are ill-prepared.

It is not here suggested that next on the agenda of health care in Africa is to devote attention and resources to training bioethics consultants for the bedside. Most people in Africa still do not have access to qualified health personnel and reasonable health care. The point is that deliberate efforts should be made to train present and future health care providers to be aware of the core moral virtues required of them in their duties to patients; and be sensitive to the ethical values of their patients, their families and the society.

### Ethics education

In the developed world education in ethics is no longer a "hidden curriculum" [17] that is passively passed to medical students during their training. It has become an "open" subject that is actively taught, not only in medical schools, but also in institutions that train other categories of health care workers. It has also become a required module in the training of resident doctors in most countries where bioethics is well grounded. Ethics education is aimed at teaching the cognitive and behavioural aspects of ethics for the purpose of improving the quality of care in terms of both the process and outcome of care. It enhances the student's ability to integrate the technical and moral components of the decision making process in clinical practice [16]. It also prepares the recipients to meet with ethical challenges of clinical practice and biomedical research. The pedagogic formats used include didactic teaching, clinical case studies, small-group discussion of ethical issues, and ethics rounds or grand rounds. A survey of medical graduates in the United States who had received ethics teaching while at medical school revealed that they were better suited to confront ethical issues in their practice and favoured continuation and expansion of ethics teaching in medical schools [18]. Other reports from both developed and developing countries that evaluated medical ethics programmes among medical students attest to the positive impact it has on their appreciation of ethical issues in clinical practice and the way they resolve them [19-22].

It is now time for Africa to join the rest of the world by introducing ethics education into the curricula of all medical schools where it is not presently taught. This is where the future of bioethics and health care delivery and research in Africa lies. Apart from some countries in the southern and eastern part of Africa and a handful of universities in other parts of Africa, there is no formal ethics education in most of Africa's medical schools. Ethics education to medical students is a necessary and required commitment to accomplishing an all-round training of the doctor whose decisions are both technical and moral. Much attention has wholly focused on the technical aspect of medical education, leaving the student to develop his or her moral attitudes passively through observation and intuition. Formal ethics teaching aims at equipping students with a common framework on which to reconcile patients' medical needs with their values, perceptions, situations and beliefs [16]; and may be a process towards achieving the hitherto elusive regulation of medical practice in most of these developing countries.

Pertinent to any discussion about teaching bioethics in Africa is the issue of shortage of trained bioethicists to fill the vacancies that would be created in academic institutions in many African countries. Apart from South Africa and a few others, most countries in Africa lack the requisite bioethics manpower that would be needed in the medical schools. Even in institutions where bioethics is already part of the medical curriculum, it is unlikely that there are enough bioethics teachers. It is in this regard that the efforts of international agencies that fund training of developing world bioethicists are noteworthy. Those Africans who have undergone bioethics training in the developed world and have become pioneers in their institutions have an awesome responsibility of establishing credible training agenda for their countries. They are also well positioned to directly seek funding for such home-based programmes from international sponsors. At the beginning, such scholars may encounter some crisis of identity and acceptance within the established academic system. However, such difficulties would fizzle out as they persist in highlighting and proffering solutions to the myriad of contemporary ethical problems within the system and the society. The initial difficulty of publishing their works in established western bioethics literature can be overcome by patronizing local or regional journals that target the primary audience for which their work is meant and open access journals that reach far and wide. Moreover, most bioethics literature consists of commentaries, observations, personal opinions and philosophical reasonings. As African boethicists embark on qualitative research to highlight ethical issues in Africa and provide quantitative data to fill the gap and provide information which are frequently lacking in most western bioethics journals, access to western dominated journals would be enhanced.

In an editorial published in a recent issue of *Bioethics*, Chadwick and Schuklenk question the altruism behind training developing world bioethicists in the west and warn against bioethics colonialism. They opine that graduates of such programmes are subjected to western ethical views and ideologies and that the developing world is not funded to develop bioethics capacity based on its own thinking [23]. While I agree with and advocate the principle that more funding should be channeled into establishing bioethics training programmes for Africa, in Africa and by Africans, as is being presently done in South Africa, I do not support the notion that those who have received bioethics training abroad are necessarily placed at the mercy of their trainers to the point of becoming their stooges and hangers-on. Though schooled in the western bioethics tradition I am of the opinion that such trainees have been given the necessary training to critically analyze ethical issues and formulate bioethical frameworks from an African perspective. Their immediate post-training thinking which appears to be shaped by western sentiments would become more and more Afro-centric as they begin to identify, appreciate and explore the hitherto unexplored and emerging ethical issues in their jurisdiction. Rather than retreiting and reinforcing western notions, there are enough ethical issues in the developing world to which such trainees could direct their searchlight and scholarship.

## Summary

In the light of the foregoing, it is imperative that African bioethics must evolve which should take cognizance of its unique needs and circumstances and which, though amenable to improvement as a result of continuing interactions with other cultures and values, yet is not overshadowed by those influences. The need for the individual clinician/researcher to be committed to upholding high ethical standards and principles that respect the social, cultural, economic, educational and religious values of the people can not be over-emphasized. More efforts are required towards increasing continent-wide awareness about ethical issues in biomedical practice and research through ethics conferences, workshops, national bioethics conferences, the public media and Non-Governmental Organisations (NGOs). Countries where bioethics presence is fairly strong should assist neighboring countries to establish a presence, especially in organizing ethics review committees at research centres and institutions. Within countries, the possibility of joint or regional ethics review committees should be explored.

Continuing and expanded support from the international bioethics community is required now more than before, to develop capacity for training of academic faculty, clinicians, researchers, government health ministry officials, NGOs, and the media in bioethics. The initiatives of the National Institute of Health of the United States to provide training grants for bioethics programmes within and outside Africa and the support of other institutions and bodies like the Wellcome Trust, African Malaria Network Trust (AMANET) and European Forum for Good Clinical Practice (EFGCP) among others, to the course of bioethics in Africa are noteworthy. Their support for bioethics capacity building programmes should not be limited to one or two sub-regions but to the whole continent. Further sponsorship should be provided for academic institutions within Africa to establish more short- and long-term training programmes at sub-regional levels. More importantly, these institutions should support the movement towards formal incorporation of bioethics into the curricula of medical schools and other health training institutions in Africa. The present and future needs for this in Africa are most apparent now. According to an African adage, the best time to plant a tree is twenty years ago, the next best time is now.

## Competing Interests

The author was a Fogarty Fellow (2003/2004) at the Joint Centre for Bioethics, University of Toronto 88, College Street, Toronto, Ontario, Canada M5G 1L4.

## Pre-publication history

The pre-publication history for this paper can be accessed here:


